# Speech Sounds Production, Narrative Skills, and Verbal Memory of Children with 22q11.2 Microdeletion

**DOI:** 10.3390/children11040489

**Published:** 2024-04-19

**Authors:** Marijana Rakonjac, Goran Cuturilo, Natasa Kovacevic-Grujicic, Ivana Simeunovic, Jovana Kostic, Milena Stevanovic, Danijela Drakulic

**Affiliations:** 1 Institute for Experimental Phonetics and Speech Pathology, Jovanova 35, 11000 Belgrade, Serbia; masadanicic@gmail.com; 2 Faculty of Medicine, University of Belgrade, Dr Subotica 8, 11000 Belgrade, Serbia; goran.cuturilo@udk.bg.ac.rs; 3 University Children’s Hospital, Tirsova 10, 11000 Belgrade, Serbia; 4 Institute of Molecular Genetics and Genetic Engineering, University of Belgrade, Vojvode Stepe 444a, 11042 Belgrade, Serbia; natasakovacevic@imgge.bg.ac.rs (N.K.-G.); ivana.simeunovic@imgge.bg.ac.rs (I.S.); jkostic@imgge.bg.ac.rs (J.K.); milenastevanovic@imgge.bg.ac.rs (M.S.); 5 Faculty of Biology, University of Belgrade, Studentski trg 16, 11000 Belgrade, Serbia; 6 Serbian Academy of Sciences and Arts, Kneza Mihaila 35, 11000 Belgrade, Serbia

**Keywords:** 22q11.2 microdeletion, articulatory characteristics of speech sounds, expressive language, receptive language, immediate verbal memory, congenital heart defects

## Abstract

22q11.2 deletion syndrome (22q11.2DS), the most frequent microdeletion syndrome in humans, is related to a high risk of developing neurodevelopmental disorders. About 95% of patients with 22q11.2DS have speech and language impairments. Global articulation, story generation, and verbal memory tests were applied to compare articulatory characteristics of speech sounds, spontaneous language abilities, and immediate verbal memory between four groups of Serbian-speaking children: patients with 22q11.2DS, children with clinical presentation of 22q11.2DS that do not have the microdeletion, children with non-syndromic congenital heart defects, and their peers with typical speech–sound development. The obtained results showed that children with this microdeletion have impaired articulation skills and expressive language abilities. However, we did not observe weaker receptive language skills and immediate verbal memory compared to healthy controls. Children with 22q11.2DS should be considered a risk category for the development of speech–sound pathology and expressive language abilities. Since speech intelligibility is an instrument of cognition and adequate peer socialization, and language impairment in school-aged children with 22q11DS might be an indicator of increased risk for later psychotic symptoms, patients with 22q11.2 microdeletion should be included in a program of early stimulation of speech–language development immediately after diagnosis is established.

## 1. Introduction

The most frequent chromosomal deletion syndrome in humans, with a prevalence of about 1:2000 live births, is 22q11.2 deletion syndrome (22q11.2DS) [1]. It is caused by a heterozygous microdeletion of the region q11.2 of chromosome 22 [2,3], and in approximately 72–94% of the patients, the deletion occurs *de novo*, while in 6 to 28% of cases, the microdeletion is inherited [4,5]. The clinical presentation of the 22q11.2DS is characterized by variable expression with over 190 clinical features associated with this syndrome [6,7]. The most common are congenital heart defects (CHD), characteristic facial appearance, thymic hypoplasia, T-cell immunodeficiency, cleft palate/velopharyngeal insufficiency, hypoparathyroidism with hypocalcemia, feeding difficulties, and high rates of psychiatric illnesses such as autism spectrum disorders, attention-deficit/hyperactivity disorder, and schizophrenia spectrum disorders [3,8,9]. Also, speech and language impairment is found in about 95% of children with 22q11DS [10]. It was shown that 80% of children at age three were non-verbal or spoke only words or simple phrases [11]. Most children with this syndrome show learning difficulties that are both verbal (e.g., speech, language, and articulation) and non-verbal (e.g., motor skills and visuospatial organization) [12]. These speech–sound disorders frequently lead to poor speech intelligibility, especially in children at a younger age [10,13,14]. Furthermore, language impairment in school-aged children with 22q11DS might be an indicator of increased risk for later psychotic symptoms [15].

There are no data in the literature about the articulatory characteristics of speech sounds, spontaneous language abilities, and immediate verbal memory of children with 22q11.2 microdeletion who are native speakers of South-Slavic languages. Thus, we aimed to analyze these characteristics in four groups of children, monolingual native speakers of the Serbian language: children with 22q11.2 microdeletion, children who have a phenotype that resembles 22q11.2DS but do not have 22q11.2 microdeletion, children with non-syndromic CHD (taking into account a report suggesting that children with non-syndromic CHD may display speech and language impairments [16]), and their age-matched peers who have proper speech and language development and good overall health.

## 2. Materials and Methods

### 2.1. Participants

Four groups of children were analyzed in this study:

(1) Group E1 comprised 15 participants with confirmed 22q11.2 microdeletion (8 males, 7 females) (Table 1 and Table S1);
(2) Group E2 consisted of 14 participants with a phenotype resembling 22q11.2DS that did not have the microdeletion (9 males, 5 females) (Table 1 and Table S1);
(3) Group E3 consisted of 14 participants with non-syndromic CHD (9 males, 5 females) (Table 1 and Table S1);
(4) Group C comprised 14 participants with typical speech–sound development, good general health, and no chronic diseases (10 males, 4 females) (Table 1 and Table S1).

Children from groups E1 and E2 had at least two out of five major phenotypic features of 22q11.2DS (CHD, facial dysmorphism, hypocalcemia, thymic hypoplasia, cleft palate; all of them had CHD) (Table S1).

Children were tested by health professionals from the University Children’s Hospital, Belgrade, Serbia, and speech–language specialists from the Institute for Experimental Phonetics and Speech Pathology, Belgrade, Serbia. Information about families’ socio-economic status was obtained through interviews. All examined children were Caucasian, 5.5–12 years old, and they were all monolingual native speakers of the Serbian language. More than 60% of children from the E1, E2, and E3 groups and all control subjects lived in cities. Also, more than 75% of parents from all analyzed groups were employed; data are missing for one child from group E1 (lived with grandparents) and one child from group E2 (lived with a foster family). More than 80% of parents from the E1 and E2 groups and 100% from the E3 and control groups finished secondary school or had a bachelor’s degree. 

The Ethical Committees of the University Children’s Hospital and Institute for Experimental Phonetics and Speech Pathology approved the study protocol. Prior to participation in this study, informed written consent was obtained from their parents.

### 2.2. FISH and MLPA

Fluorescence in situ hybridization (FISH) or multiplex ligation-dependent probe amplification (MLPA) analysis were applied for the detection of 22q11.2 microdeletion, as described by Cuturilo et al. [17]. FISH analysis was carried out on metaphase spreads from cultivated lymphocytes using the probe that spans the common deletion region (TUPLE1, 22q11.2, SpectrumOrange) and the control probe (ARSA, 22q13.3, SpectrumGreen) (Abbott Molecular Inc., Des Plaines, IL, USA). To confirm or exclude the 22q11.2 microdeletion, at least 30 metaphases were scored. 

### 2.3. Assessment of Articulatory Characteristics of Speech Sounds

To analyze the articulatory characteristics of speech sounds in children from four groups (E1, E2, E3, and C), the global articulation test was applied as described by Rakonjac et al. [18]. Briefly, the quality of the pronounced phonemes was scored as follows: scores from 1 to 3 were given to phonemes pronounced according to the standard norm for the Serbian language; marginally pronounced phonemes were scored by 4; distorted phonemes were scored by 5 and 6; and phonemes substituted with other phonemes (pathological substitutions) or omissions (not pronounced phonemes) were scored by 7. The examination was carried out in a quiet room individually. The speech–language therapist introduced herself to the participant and described how the testing would be performed. The child was asked to provide consent before the testing, and appropriate breaks were given when the child needed them.

### 2.4. Assessment of Spontaneous Language Abilities 

To analyze the spontaneous language abilities of children from all groups, a story generation test was applied as described by Rakonjac et al. [18]. The spontaneous language abilities were analyzed by an evaluation of the child’s ability to produce a story from a set of four images [19]; the spontaneous speech was then recorded and transcribed orthographically. In this study, the total number of words, the total number of grammatical and ungrammatical sentences (clauses), and the number of grammatical clauses were observed.

### 2.5. Assessment of Immediate Verbal Memory

A verbal memory test was used for the evaluation of immediate verbal memory. In the test, there were 55 verbal stimuli divided, according to the difficulty of the request, into 6 groups (the first group contained syllables, the second group contained two-syllable words, the third group contained two-syllable nonsense words, the fourth group contained simple sentences, the fifth group contained compound sentences, and the sixth group contained complex sentences). Groups 1 to 5 contained 10 verbal stimuli each, while the sixth group contained 5 verbal stimuli. Based on defined norms, children between six and seven years of age should reproduce without delay all verbal stimuli in the first five groups.

An assessment of immediate verbal memory was performed by asking the subject to immediately repeat a given verbal stimulus. The quality of voice pronunciation (articulation) was neglected. A word or sentence was considered complete if it had the appropriate number of syllables or the completeness of the composition, respectively. Each correct answer was marked with one point. If the sentence was not repeated verbatim, it was marked 0. 

### 2.6. Statistics 

Statistical analysis was performed in R, version 4.3.2 [20]. The normality of the data was tested with the Shapiro–Wilk test. In cases where the Shapiro–Wilk test did not indicate normal distribution (the articulatory characteristics of speech sounds, the number of pronounced phonemes, the number of produced sentences, grammatically correct sentences, and reproduced verbal stimuli), the data were tested with a non-parametric Kruskal–Wallis test. A post hoc Dunn test with Bonferroni correction was used to reveal differences between pairs of groups.

Differences in the number of produced words were tested with a one-way ANOVA, followed by a Tukey post hoc test for pairwise comparisons between groups since the data showed a normal distribution.

Fisher’s exact test was performed to examine if there was an association between the groups of patients and their ability to understand the linguistic content of the shown images. For all analyses, a *p* value < 0.05 was considered significant.

## 3. Results

### 3.1. Detection of 22q11.2 Microdeletion

The presence of 22q11.2 microdeletion was confirmed in 13 patients from group E1 and excluded in 13 patients from group E2 during our previous studies (Table S1) [18,21,22]. In this study, we analyzed the presence of 22q11.2 microdeletion by FISH and/or MLPA in two patients from group E1, one patient from group E2, and all patients from group E3 (Table S1). Microdeletion was detected only in two patients from group E1. A familial form of 22q11.2 microdeletion was found in three tested families (patients no. 15, 31, and 40, Table S1). In all these cases, the microdeletion was inherited maternally. The mother of patient no. 15 exhibited nasal speech and borderline intellectual functioning, while speech and language impairments were not detected in the mothers of patients no. 31 and 40. 

### 3.2. Analysis of Articulatory Characteristics of Speech Sounds in Children with 22q11.2 Microdeletion

**Figure 1:**
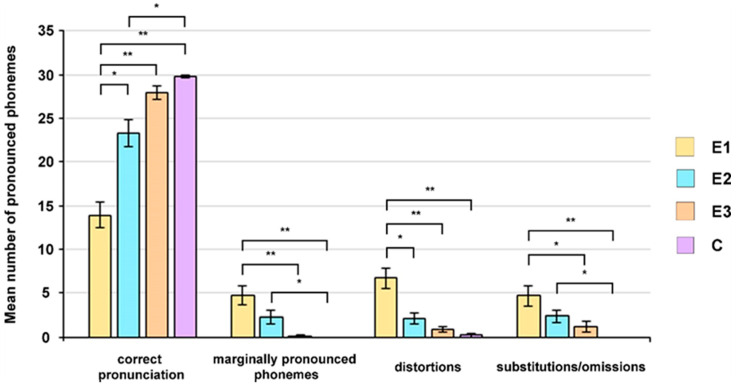
Articulatory characteristics of the phonemes of patients belonging to the E1, E2, and E3 groups and their peers from the control group using the global articulation test. Results are presented as the mean ± standard error of the mean (SEM). Differences between the groups were estimated by a Dunn–Bonferroni post hoc analysis (* *p* < 0.05, ** *p* < 0.001).

To detect if 22q11.2 microdeletion influences the articulation skills of microdeletion carriers, a global articulation test was employed, and the pronunciation of phonemes was compared between the children from all groups (Figure 1). In our previous work, which included 11 children from groups E1, E2, and C, we detected more misarticulated phonemes in group E1 than in the E2 and C groups, and in group E2 compared to group C [21]. Here we performed a detailed analysis of the articulatory characteristics of speech sounds in children from all four groups and observed that the average number of adequately pronounced sounds in patients from group E1 was lower compared to the values obtained for their peers from the other three groups (Figure 1). The Kruskal–Wallis test showed that the detected differences between the groups were statistically significant (*p* < 0.001), and the post hoc Dunn test with Bonferroni correction revealed significant differences in the number of correctly pronounced phonemes between patients from group E1 and their peers from groups E2, E3, and C, as well as between groups E2 and C (Figure 1).

The global articulation test also revealed that children belonging to groups E1 and E2 more frequently pronounced phonemes marginally compared to their peers from the E3 and control groups (Figure 1). The results of the Kruskal–Wallis test indicated statistically significant between-group differences (*p* < 0.001), and the Dunn–Bonferroni’s post hoc test showed statistically significant differences in the number of marginally pronounced phonemes between patients from group E1 and subjects from groups E3 and C, as well as between groups E2 and C (Figure 1).

Analyzed groups of children also differ in the average number of pathologically distorted sounds, with the group of 22q11.2del carriers (E1) showing the poorest performance (Figure 1). Statistically significant differences between groups (*p* < 0.001) were revealed by the Kruskal–Wallis test. The Dunn–Bonferroni’s test showed statistically significant differences in the number of pathological distortions between patients from group E1 and their peers from groups E2, E3, and C (Figure 1). 

The global articulation test revealed a higher number of pathologically substituted or omitted phonemes in patients from groups E1 and E2 compared to the values obtained for their peers from the E3 and control groups (Figure 1). Statistically significant differences were found between groups (Kruskal–Wallis, *p* < 0.001). The Dunn–Bonferroni’s test showed statistically significant differences in the number of substitutions and omissions between patients from groups E1 and E2 and control group subjects, and between groups E1 and E3 (Figure 1). 

Patients belonging to groups E1 (22q11.2 microdeletion carriers) and E2 (non-carriers with phenotypes resembling 22q11.2DS) had less developed articulation abilities compared to their peers from the control and E3 groups (children with non-syndromic CHD). In addition, a more severe degree of articulation disorder was observed in patients from group E1 compared to their peers from group E2. Namely, in group E1, there were no children adequately pronouncing all phonemes (Table S2). In group E2, about 14% of children adequately pronounced all phonemes. On the other hand, about 50% and 85% of children from groups E3 and C adequately pronounced all phonemes, respectively. Also, patients from group E1 more frequently pathologically pronounced phonemes from four groups (laterals, fricatives, affricates, and vibrant R〈r〉), while patients from groups E2 frequently pathologically pronounced laterals, affricates, and vibrant R〈r〉; children from group E3 pathologically pronounced laterals and vibrant R〈r〉). The majority of the children from the control group (C) adequately pronounced all phonemes. 

#### 3.2.1. Pronunciation of Vowels

Adequate pronunciation of all vowels was found in 46% of patients from the E1 group, about 93% of patients from the E2 group, and 100% of their peers from the E3 and C groups (Table S2). Marginal pronunciation of all vowels was observed in 33% of children from group E1 and about 7% of children from group E2. Distorted vowels were detected only in one child (6.67%) from group E1. Marginal pronunciation of the vowel A〈a〉 was detected in one child from group E1 (13.33%), whereas another child from this group marginally pronounced two vowels (O〈o〉, U〈u〉). The Kruskal–Wallis test showed statistically significant between-group differences in the pronunciation of vowels (*p* < 0.001). Pairwise comparisons using the Dunn–Bonferroni test indicated that group E1 shows a statistically significant difference when compared with groups E2, E3, and C. 

#### 3.2.2. Pronunciation of Plosives

**Figure 2:**
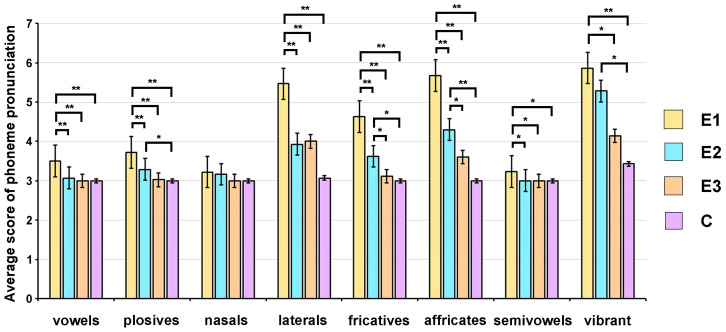
Pronunciation of phoneme groups by patients from groups E1, E2, and E3 and their peers from the control group using the global articulation test. Results are presented as the mean ± standard error of the mean (SEM). Differences between the groups were estimated by a Dunn–Bonferroni post hoc analysis (* *p* < 0.05, ** *p* < 0.001).

Only three children (20%) from group E1 adequately pronounced all plosives, compared to 64%, 93%, and 100% of their peers from groups E2, E3, and C, respectively (Table S2). Pathological pronunciation (marked from 5 to 7) of plosives P〈p〉, U〈u〉, K〈k〉, and G〈g〉 was detected only in patients from groups E1 (6.7%, 6.7%, 6.7%, 13.3%, respectively) and E2 (14.3%, 7.1%, 7.1%, 7.1%, respectively). On the other hand, only patients from group E1 pathologically pronounced T〈t〉 (33.3%) and D〈d〉 (40%). The Kruskal–Wallis test indicated statistically significant between-group differences (*p* < 0.001). The Dunn–Bonferroni post hoc test showed statistically significant differences between patients from group E1 and their peers from the other three groups and between children from the E2 and C groups (Figure 2).

#### 3.2.3. Pronunciation of Nasals

Adequate pronunciation of all nasals was revealed in 80% and 85.7% of patients from groups E1 and E2, respectively, and in all children from groups E3 and C (Table S2). There were no statistically significant differences between the examined groups in the pronunciation of nasals (Figure 2). Pathologically pronounced nasal in groups E1 (20%) and E2 (7.1%) was Nj〈ɲ〉. 

#### 3.2.4. Pronunciation of Laterals

Satisfactory pronunciation of all laterals was detected in 13.3% of children from group E1, compared to about 57%, 64%, and 93% of children from the E2, E3, and C groups, respectively (Table S2). The Kruskal–Wallis test indicated statistically significant between-group differences (*p* < 0.001), while the Dunn–Bonferroni comparison revealed significant differences in pronunciation of laterals between patients from group E1 and their peers from groups E2, E3, and C (Figure 2). Lateral L〈l〉 was pathologically pronounced in about 47% of children from group E1 and 14% of children from groups E2 and E3, while all control group subjects had adequate pronunciation of this lateral (Table S2). On the other hand, lateral Lj〈ʎ〉 was pathologically pronounced in 80% of patients from group E1, about 36% of patients from groups E2 and E3, and about 7% of children from group C (Table S2).

#### 3.2.5. Pronunciation of Fricatives

Adequate pronunciation of all fricatives was detected in only 13.3% of patients from group E1, compared to 50%, 86%, and 100% of participants from the E2, E3, and C groups, respectively (Table S2). Omitted or substituted fricatives were only detected in groups E1 and E2. Pathological pronunciation of fricative F〈f〉 was detected only in group E1. In group E3, only pathological pronunciation of fricatives Š〈∫〉 and Ž〈ʒ〉 was detected (about 14% of patients) (Table S2). The Kruskal–Wallis test indicated statistically significant between-group differences (*p* < 0.001). A post hoc test showed that group E1 scores were significantly different from scores obtained for groups E2, E3, and C (Figure 2). Significant differences were also found between groups E2 and E3, and E2 and C (Figure 2). 

#### 3.2.6. Pronunciation of Affricates

Satisfactory pronunciation of all affricates was detected in about 7% of patients from group E1, compared to about 28% and 64% of their peers from groups E2 and E3, respectively (Table S2). All the children from the control group adequately pronounced affricates (Table S2). The Kruskal–Wallis test indicated significant between-group differences in the pronunciation of affricates (*p* < 0.001). Pairwise comparisons using the Dunn–Bonferroni test indicated statistically significant differences between patients from group E1 and their peers from groups E2, E3, and C (Figure 2). Also, scores for group E2 were significantly different from the values obtained for groups E3 and C (Figure 2).

#### 3.2.7. Pronunciation of Semivowels

Pathological pronunciation of semivowels was detected only in patients from group E1 (about 26% of children) (Table S2). The Kruskal–Wallis test indicated significant between-group differences (*p* < 0.01). Pairwise comparisons using the Dunn–Bonferroni test showed statistically significant differences in the pronunciation of semivowels between patients from group E1 and children from groups E2, E3, and C (Figure 2). 

#### 3.2.8. Pronunciation of Vibrant

Pathological pronunciation of vibrant R was detected in about 93% of patients from group E1, compared to about 64%, 28%, and 14% of children from the E2, E3, and control groups, respectively (Table S2). The Kruskal–Wallis test showed significant between-group differences (*p* < 0.001). The Dunn–Bonferroni comparison revealed significant differences in the pronunciation of vibrant between groups E1 and E3, E1 and C, and E2 and C (Figure 2). 

### 3.3. Analysis of Spontaneous Language Abilities 

**Figure 3:**
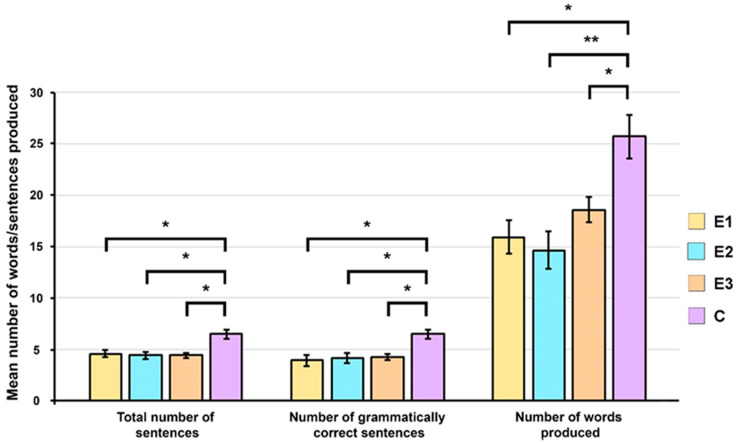
Analysis of spontaneous language skills of 22q11.2DS children (E1), their peers with 22q11.2DS clinical presentation that do not have 22q11.2 microdeletion (E2), children with non-syndromic CHD (E3), and children with typical speech–sound development (C). Differences between the groups were assessed by a post hoc analysis (Dunn–Bonferroni or Tukey tests, * *p* < 0.05, ** *p* < 0.001).

The expressive and receptive speech of participants from groups E1, E2, E3, and C was evaluated using a story generation test. The analysis of the number of sentences produced when describing the events in the images showed that the patients from groups E1, E2, and E3 produced a smaller number of sentences compared to the subjects from group C (Figure 3). The Kruskal–Wallis test indicated that the differences between the groups were statistically significant (*p* < 0.01). The post hoc Dunn–Bonferroni test showed that there were statistically significant differences in the total number of sentences produced between the patients from groups E1, E2, and E3 and control group subjects, while there were no statistically significant differences among groups E1, E2, and E3 (Figure 3).

Patients from groups E1, E2, and E3 produced a smaller number of grammatically correct sentences compared to the control subjects (Figure 3). The Kruskal–Wallis test showed that the differences between the groups were statistically significant (*p* < 0.01). The Dunn–Bonferroni’s post hoc test revealed statistically significant differences in the number of grammatically correct sentences produced between the patients from groups E1, E2, and E3 and children from group C, while statistically significant differences were not observed between the patients from the E1, E2, and E3 groups (Figure 3).

During the analysis of the total number of words produced when describing the events in the pictures, it was observed that the patients from groups E1, E2, and E3 produced a smaller number of words compared to the subjects from group C (Figure 3). The one-way ANOVA showed statistically significant differences between the groups (*p* < 0.001). A Tukey post hoc test revealed that the differences in the total number of words produced between patients belonging to groups E1, E2, and E3 and subjects from the control group were statistically significant (Figure 3). 

The next step in the analysis included the assessment of the receptive language abilities (understanding of the linguistic content of the images from the story generation test) of patients from the E1, E2, and E3 groups and control subjects. Among the analyzed groups, a Fisher’s exact test showed no statistically significant differences in the development of the receptive language abilities of patients belonging to groups E1, E2, and E3 compared to the control group subjects (Table 2). 

### 3.4. Assessment of Immediate Verbal Memory 

**Figure 4:**
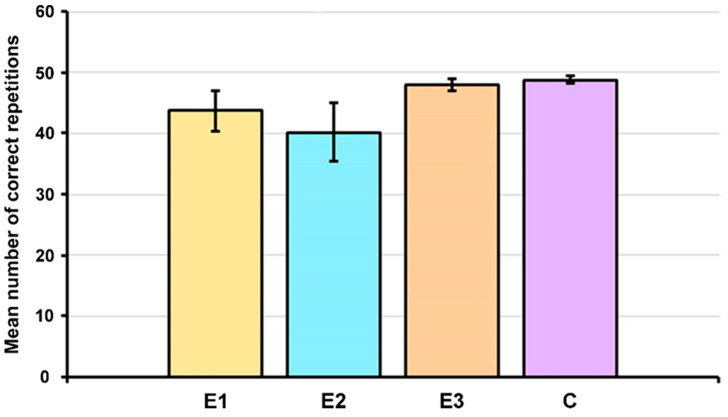
Average number of reproduced stimuli by patients from the E1, E2, and E3 groups and control subjects (C) during the verbal memory test. Results are presented as the mean ± standard error of the mean (SEM). Differences between the groups were estimated by a Kruskal–Wallis test.

The next step in the investigation of the speech and language abilities of patients from the E1, E2, and E3 groups and control subjects included the assessment of immediate verbal memory using the verbal memory test. There were no statistically significant differences in the number of correct repetitions between the analyzed groups of children (Figure 4), indicating their similar ability to use verbal memory.

## 4. Discussion

Here we analyzed the articulatory characteristics of speech sounds, expressive and receptive language, and immediate verbal memory of patients with microdeletion 22q11.2 in native speakers of the Serbian language. In a previous study, we revealed a delay in first functional word production, a higher number of misarticulated phonemes, a deficit in oral praxis, and a delay in speech and language development (using the scale for evaluation of psychophysiological abilities) in children with 22q11.2 microdeletion compared to children with the phenotype resembling 22q11.2DS who do not have microdeletion and control subjects [21] (Table S3). This study represents the continuation of our work, with a more detailed analysis of articulation skills, receptive and expressive language abilities, and immediate verbal memory in Serbian-speaking children with 22q11.2 microdeletion. We showed that children with 22q11.2DS have impaired articulation skills and expressive language abilities. On the other hand, no statistically significant differences in receptive abilities and immediate verbal memory were detected between 22q11.2DS children and their peers from E2, E3, and C groups. 

The number of studies that have analyzed the articulation skills of children with 22q11.2 microdeletion is limited. We found that the articulation skills of 22q11.2 microdeletion carriers are less developed when compared to the skills of their peers from the other analyzed groups. They have fewer correctly pronounced phonemes and a higher number of distortions compared to their peers from the other three groups, as well as a higher number of marginally pronounced phonemes and substitutions/omissions compared to children from groups E3 and C. Only in the group of children with 22q11.2DS were there no patients who adequately pronounced all phonemes. Our results are in line with previous research indicating that articulation disorders occur in 77% of patients with 22q11.2DS [14]. Also, 22q11.2 microdeletion carriers have impaired speech intelligibility due to a large number of inadequately pronounced phonemes [11,14,23] (Table S3). One of the reasons for the lower articulation skills of patients with 22q11.2DS could be a deficit in terms of fine motor development and the performance of coordinated locomotor models. In our previous report, we showed that motor skills were less developed in children from group E1 compared to their peers from groups E2 and C [21]. Also, the reason for the lower articulation skills of patients from group E1 might be a lower level of their speech and language development. Previously, we found that the speech and language development of children from group E1 lagged behind that of children from groups E2 and C [21]. Although it has been reported that usually children with congenital heart disease have an impairment of speech and language [16,24], we did not detect statistically significant differences in the number of correctly and marginally pronounced phonemes, the number of distortions, and substitutions/omissions between groups E3 and C. The case of monozygotic twins with 22q11.2DS and hypernasal speech, in which only one of them had congenital heart disease [25], suggests that hypernasal speech in 22q11.2 microdeletion carriers is not exclusively due to CHD but that other factors contribute to this articulation disorder. Furthermore, we found less developed articulation skills in children from group E2 compared to the control group subjects. Since we did not detect differences in articulation abilities between children from groups E3 and C, we can postulate that factors other than CHD contribute to the impairment of articulation skills in children from group E2. Although the differences in articulation skills between the patients from the E2 and E3 groups are not statistically significant, the average number of correctly pronounced phonemes is lower, and the average numbers of marginally pronounced, distorted, and substituted or omitted sounds are higher in children from the E2 group than in children from the E3 group. Research that will include a larger number of patients will enable a more precise determination of whether there is a difference in articulation abilities between patients with syndromic and non-syndromic CHD. Furthermore, we found the pathological pronunciation of fricatives only in children from group E1. In addition to fricatives, children from group E1 frequently pathologically pronounced laterals, affricates, and vibrant R〈r〉. This is in line with previously published data revealing that children with 22q11.2DS, speakers of the Spanish language, have major difficulties in the pronunciation of fricatives, affricates, and a vibrant [26] (Table S3). The most misarticulated consonants in 22q11.2DS patients, speakers of the Swedish language, were stops and fricatives [13] (Table S3).

Usually, delays in expressive language are revealed in patients with 22q11.2DS [10,14,27,28,29,30] (Table S3). A smaller number of sentences, grammatically correct sentences, and produced words when generating a story from a set of four pictures was detected in patients from groups E1, E2, and E3 compared to the control group subjects. This is in concordance with a previous study showing that 22q11.2DS patients have difficulties retelling a narrative task. Also, reduced sentence length and grammatical complexity and a low prevalence of grammatical errors were found in patients with 22q11.2DS [31] (Table S3). Weak expressive grammar was detected in children with 22q11.2DS [32], and school-aged children with this syndrome have difficulties with word learning [14,31] (Table S3). Shorter, less grammatically complex sentences were revealed in English- and Dutch-speaking children with 22q11.2 microdeletion compared to typically developing children [33] (Table S3). Detected differences may be a consequence of a lack of object recognition in the picture. The results of the previous research indicated impaired visual perception and processing capabilities in children with 22q11.2DS [34]. However, we did not detect statistically significant differences in the number of produced sentences, grammatically correct sentences, and words between groups E1, E2, and E3. We can postulate that CHD contributes to lower levels of expressive language abilities in children from these three groups. In parallel with this, the literature data indicate that narrative discourse may need special attention in children who undergo corrective cardiac surgery as infants [35].

A previous study showed that weaker receptive language abilities in children with 22q11DS are associated with increased behavioral problems in social communication and interaction [36]. In the literature, there are opposing data regarding the receptive language skills of patients with 22q11.2DS. While Glaser et al. found that receptive language abilities are lower than expressive ones [37], other authors revealed that expressive language skills are lower than receptive ones [11,29]. Also, the literature data revealed that both expressive and receptive language abilities of 22q11DS children are significantly weaker than in children with typical language development [29,38]. We did not detect statistically significant differences in the receptive language abilities between children from all four analyzed groups. This is in concordance with the report of Kambanaros et al., who did not detect differences in receptive language abilities between a Greek-speaking child with 22q11DS and his peers with typical language development [39] (Table S3). Also, Roizen et al. did not find differences in receptive language skills between children with 22q11.2DS and community and sibling controls [28]. We did not detect statistically significant differences in receptive language skills between children from groups E3 and C. However, the literature data suggest that patients with CHD have reduced receptive language skills after corrective surgery for Tetralogy of Fallot or ventricular septal defect in infancy [40].

We did not detect statistically significant differences in immediate verbal memory between children from groups E1, E2, E3, and C. This is in line with the results obtained by other authors [41,42,43], although the literature data also pointed to poorer verbal memory in patients with 22q11.2DS [44,45,46] (Table S3). It would be of interest to analyze the long-term verbal memory of children from all four groups since the literature data revealed a deficit in long-term verbal memory in patients with 22q11.2DS when delays longer than 30 min were examined [47].

The limitation of our study is the small sample size investigated compared to the majority of previous data regarding 22q11.2DS. However, the Republic of Serbia is a small country with about 6.7 million inhabitants, and the detection of 22q11.2 microdeletion started in 2003. Also, due to congenital heart defects, some of the patients with this microdeletion died before they were 5.5 years old. Also, only one center contributed to the data collection.

## 5. Conclusions

Serbian-speaking children with 22q11.2DS have impaired articulation skills and expressive language abilities. On the other hand, we did not detect that receptive language abilities and immediate verbal memory were reduced in these patients. Altogether, children with 22q11.2DS should be considered a risk category for the development of speech–sound pathology and expressive language abilities and should be included in a program of early stimulation of speech–language development as soon as a diagnosis is established.

## Figures and Tables

**Table 1:** Description of the groups of children analyzed in this study.

Group	No. of Patients	22q11.2 Deletion	22q11.2DS Phenotype	Non-Syndromic CHD
**E1**	15	yes	yes	no
**E2**	14	no	yes	no
**E3**	14	no	no	yes
**C**	14	no	no	no

**Table 2:** Analysis of the receptive language abilities of patients from the E1, E2, and E3 groups and control group subjects. The number of children from each group that understood or did not understand the content of the images in the story generation test is indicated.

Group	Understood the Content	Did Not Understand the Content	Total
**E1**	11	4	15
**E2**	12	2	14
**E3**	14	0	14
**C**	14	0	14
**Total**	51	6	57

## Data Availability

The data presented in this study are available on request from the corresponding author due to privacy and ethical reasons.

## Supplementary Materials

The following supporting information can be downloaded at: https://www.mdpi.com/article/10.3390/children11040489/s1, Table S1: Pronunciation of phonemes in E1, E2, E3, and C groups. Table S2: Description of the children from groups E1, E2, E3, and C. Table S3: Summary of publications on speech, language, and memory abilities of children with 22q11.2DS.
