# Supplementary material for: Speech Sounds Production, Narrative Skills, and Verbal Memory of Children with 22q11.2 Microdeletion
Source: Children (Basel). 2024 Apr 19;11(4):489. doi: 10.3390/children11040489 (PMC11049265; doi:10.3390/children11040489)
Supplement: Supplementary file 1 [file children-11-00489-s001.zip › Table S1.pdf]

Table S1. Description of the children from groups E1, E2, E3 and C

No.	No. of patient	Age (years)	Sex	Presence of 22q11.2 microdeletion	CHM	Facial dysmorphism	Hypocalcemia	Thymic hypoplasia	Cleft palate	Reference
**E1**										
**1**	**1**	6.5	M	+	+	+	+	-	-	[21,22]
**2**	**3**	6.5	M	+	+	+	-	-	-	[21,22]
**3**	**4**	9	M	+	+	-	+	-	-	[21,22]
**4**	**6**	5.5	M	+	+	-	+	-	-	[21,22]
**5**	**7**	9	M	+	+	+	-	-	-	[21,22]
**6**	**11**	6.5	F	+	+	+	+	+	-	[21,22]
**7**	**12**	5.5	M	+	+	-	-	+	-	[21,22]
**8**	**13**	6	F	+	+	-	-	+	-	[21,22]
**9**	**14**	9.5	M	+	+	+	+	-	-	[21,22]
**10**	**15**	8	F	+	+	+	-	-	-	[18,22]
**11**	**31**	7.5	M	+	+	+	+	-	-	[18,22]
**12**	**33**	9.5	F	+	+	+	+	+	-	[21]
**13**	**35**	6	F	+	+	-	-	+	-	[22]
**14**	**40**	7.5	F	+	+	+	+	-	-	this study
**15**	**60**	6	F	+	+	+	-	-	-	this study
**E2**										
**1**	**2**	6	M	-	+	+	-	-	-	[21,22]
**2**	**16**	10	M	-	+	+	-	+	-	[21,22]
**3**	**17**	5.5	M	-	+	+	-	-	-	[21,22]
**4**	**19**	10	M	-	+	+	-	-	-	[21,22]
**5**	**21**	6	F	-	+	+	-	+	-	[21]
**6**	**22**	9	F	-	+	+	-	-	-	[21,22]
**7**	**23**	8	M	-	+	+	-	+	-	[22]
**8**	**25**	6	M	-	+	+	not available	+	-	[21]
**9**	**26**	6	M	-	+	+	-	-	-	[21,22]
**10**	**27**	7	M	-	+	+	+	-	-	[21]
**11**	**28**	6.5	M	-	+	+	-	-	-	[21]
**12**	**32**	5.5	F	-	+	+	+	-	-	[21]
**13**	**41**	9	F	-	+	+	-	-	-	[22]

**14**	**64**	5.5	F	-	+	+	-	-	+	this study
**E3**										
**1**	**44**	10	M	-	+	-	-	-	-	this study
**2**	**45**	11	F	-	+	-	-	-	-	this study
**3**	**47**	7	M	-	+	-	-	-	-	this study
**4**	**49**	11.5	M	-	+	-	-	-	-	this study
**5**	**51**	6	M	-	+	-	-	-	-	this study
**6**	**29**	6.5	M	-	+	-	-	-	-	this study
**7**	**34**	10	M	-	+	-	-	-	-	this study
**8**	**53**	6	F	-	+	-	-	-	-	this study
**9**	**54**	10.5	M	-	+	-	-	-	-	this study
**10**	**56**	5.5	F	-	+	-	-	-	-	this study
**11**	**57**	8.5	F	-	+	-	-	-	-	this study
**12**	**58**	10	F	-	+	-	-	-	-	this study
**13**	**61**	12	M	-	+	-	-	-	-	this study
**14**	**62**	7	M	-	+	-	-	-	-	this study
**C**										
**1**	**C1**	8	F	-	-	-	-	-	-	[21]
**2**	**C2**	10	M	-	-	-	-	-	-	[21]
**3**	**C3**	9.5	M	-	-	-	-	-	-	[21]
**4**	**C4**	7.5	F	-	-	-	-	-	-	[21]
**5**	**C5**	7.5	F	-	-	-	-	-	-	[21]
**6**	**C7**	6.5	M	-	-	-	-	-	-	[21]
**7**	**C10**	5.5	M	-	-	-	-	-	-	[21]
**8**	**C11**	7	F	-	-	-	-	-	-	[21]
**9**	**C14**	6	M	-	-	-	-	-	-	[21]
**10**	**C16**	6	M	-	-	-	-	-	-	[21]
**11**	**C17**	6	M	-	-	-	-	-	-	[21]
**12**	**C20**	6	M	-	-	-	-	-	-	this study
**13**	**C21**	6	M	-	-	-	-	-	-	this study
**14**	**C22**	5.5	M	-	-	-	-	-	-	this study
