# Supplementary material for: Speech Sounds Production, Narrative Skills, and Verbal Memory of Children with 22q11.2 Microdeletion
Source: Children (Basel). 2024 Apr 19;11(4):489. doi: 10.3390/children11040489 (PMC11049265; doi:10.3390/children11040489)
Supplement: Supplementary file 1 [file children-11-00489-s001.zip › Table S3.pdf]

Table S3. Summary of publications on speech, language, and memory abilities of children with 22q11.2DS

Language studied	Articulation skills	Expressive language abilities	Receptive language abilities	Verbal memory	Reference
Serbian	Lower number of adequately pronounced sounds; Higher number of marginally pronounced phonemes, pathologically distorted sounds and pathologically substituted or omitted phonemes; Pathologically pronounced phonemes from 4 groups (laterals, fricatives, affricates and vibrant R⟨r⟩)	Lower expressive language abilities (smaller number of sentences, grammatically correct sentences and words produced when describing the events in the images)	Not affected	Immediate verbal memory not affected	This study
Serbian	Higher number of misarticulated phonemes; Deficit in oral praxis	Delay in production of the first word; Delay in language development	NA	NA	[21]
English	Limited consonant inventories of young children	Impaired	Impaired	NA	[27]
English	Impaired	Language difficulties in diverse linguistic domains such as syntax, vocabulary, concepts, word finding, and discourse organization	Delayed	NA	[14]
English	Persistent articulation and resonance disorders; Voice impairment	Lower scores on expressive language skills	NA	NA	[11]
English	Smaller consonant inventories; Higher	NA	NA	NA	[23]

	number of developmental errors; Higher frequency of glottal stop use				
English	Disturbed voice quality, low facial tone, articulation errors	Delayed	Delayed	NA	[38]
English	NA	Impaired	Impaired	NA	[37]
Swedish	Impaired (stops and fricatives were the most misarticulated consonants)	NA	NA	NA	[13]
English	NA	NA	NA	Poor performance on verbal working memory test	[43]
English	NA	Poor expressive language performance	NA	Poor verbal working memory	[45]
Swedish	NA	Lower narrative retell abilities (difficulties in conveying information, short sentences with low grammatical complexity)	Low receptive language scores	NA	[31]
English	NA	Delay in expressive language milestones	Not affected	NA	[28]
French	NA	NA	NA	Impaired performance on the serial order short-term memory tasks	[44]
English	NA	NA	NA	Impaired verbal memory	[41]
Greek	NA	Comparable at age 6 and lower at age 10	Not affected	NA	[39]

English/ Dutch	NA	Shorter and less grammatically complex sentences	NA	NA	[33]
Dutch	NA	NA	Word comprehension slightly to severely weakened in relation to chronological age norms; Difficulties with comprehension at the sentence level	Not affected	[42]
Spanish	Impaired (difficulties in the articulation of fricatives, affricates and vibrant rhotic consonant clusters + /r/)	NA	NA	NA	[26]
Dutch	NA	Impaired	NA	NA	[29]
French	NA	Poor performance on verbal learning	NA	NA	[46]

NA – not analyzed
